# Elevated exosome-derived miRNAs predict osimertinib resistance in non-small cell lung cancer

**DOI:** 10.1186/s12935-021-02075-8

**Published:** 2021-08-14

**Authors:** Xinying Li, Cen Chen, Zimu Wang, Jiaxin Liu, Wei Sun, Kaikai Shen, Yanling Lv, Suhua Zhu, Ping Zhan, Tangfeng Lv, Yong Song

**Affiliations:** 1 Department of Respiratory and Critical Care Medicine, Jinling Hospital, Nanjing University School of Medicine, 305 East Zhongshan Road, Nanjing, 210002 Jiangsu China; 2 Department of Respiratory and Critical Care Medicine, Nanjing Drum Tower Hospital, Nanjing University School of Medicine, Nanjing, China; 3 Department of Respiratory and Critical Care Medicine, Jinling Hospital, The first School of Clinical Medicine, Southern Medical University (Guangzhou), Nanjing, China; 4 Department of Respiratory and Critical Care Medicine, Jinling Hospital, Medical School of Southeast University, Nanjing, China; 5 Nanjing University Institute of Respiratory Medicine, Nanjing, China

**Keywords:** Osimertinib (AZD9291), Exosome, miRNAs, Bypass pathway, NSCLC

## Abstract

**Background:**

Non-small cell lung cancer (NSCLC) patients with epidermal growth factor receptor (EGFR) mutations will inevitably develop drug resistance after being treated with the third-generation EGFR-tyrosine kinase inhibitor (TKI), osimertinib. Recently, the drug resistance information transmitted by exosomal miRNAs has attracted much attention. However, the mechanism of exosome-derived miRNAs in osimertinib resistance remains unexplored.

**Methods:**

We extracted and sequenced exosomes from the supernatant of the osimertinib-resistant cell line, H1975-OR, and the sensitive cell line, H1975. The results were compared with plasma exosome sequencing before and after the appearance of drug resistance in three NSCLC clinical patients treated with oral osimertinib. Exosome-derived miRNAs that had significantly increased expression levels after osimertinib resistance were screened for expanded validation in other 64 NSCLC patients.

**Results:**

Cluster analysis of the target genes revealed that exosomal miRNAs participate in osimertinib resistance mechanisms through the activation of bypass pathways (RAS-MAPK pathway abnormality and PI3K pathway activation). Exosome-derived miR-184 and miR-3913-5p expression levels increased significantly after the onset of osimertinib resistance. Exosomal miR-3913-5p was associated with TNM stage, platelet count, tumor marker carcinoembryonic antigen, and distant metastases. In patients with EGFR exon 21 L858R mutation, the increased expression levels of miR-184 and miR-3913-5p derived from serum exosomes indicated osimertinib resistance. Similarly, for T790M-positive patients, the level of exosome-derived miR-3913-5p can be used as a predictive marker for osimertinib resistance.

**Conclusions:**

The expression levels of miR-184 and miR-3913-5p derived from exosomes in the peripheral blood of NSCLC patients could be used as biomarkers to indicate osimertinib resistance.

**Graphic Abstract:**

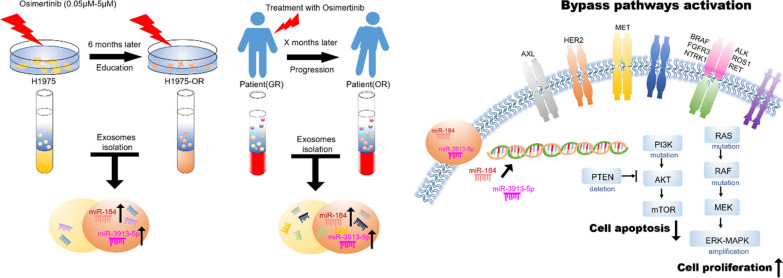

**Supplementary Information:**

The online version contains supplementary material available at 10.1186/s12935-021-02075-8.

## Background

Epidermal growth factor receptor (EGFR) mutations are the most common genetic mutations in non-small cell lung cancer (NSCLC) [1]. The PIONEER study (NCT01185314) [2] showed that the EGFR mutation rate in the Chinese population reached 50.2%. Among them, exon 19 deletion (48.9%) and exon 21 L858R point mutation (45.4%) were the most common [2, 3]. Usually, 50–60% of the NSCLC patients with EGFR mutations develop acquired resistance to tyrosine kinase inhibitors (TKI) after first- and second-generation EGFR-TKI therapy [4], especially those with p.Thr790Met (T790M) point mutation in exon 20. The FLAURA study, which received wide attention at the 2019 European Society for Medical Oncology (ESMO) congress, confirmed that EGFR-mutant NSCLC patients treated with the third-generation EGFR-TKI osimertinib [5] had a 6.8 months prolongation of overall survival (OS) compared to patients receiving gefitinib or erlotinib therapy [6]. However, patients treated with osimertinib inevitably develop resistance.

Exosomes [7] are extracellular vesicles measuring 30–150 nm that transmit biological information and participate in cell-mediated biological activities by releasing large amounts of proteins, RNA, lipids, and other biomolecules to the extracellular environment [8]. MicroRNAs (miRNAs) [9] in exosomes are not digested by RNases, allowing them to be transported between cells and participate in epigenetics [10]. Existing literatures have shown that exosomal miRNAs (exo-miRNAs) secreted by tumor cells play an important role in drug resistance [11, 12]. Whether exosomes are involved in resistance to the third-generation EGFR-TKI osimertinib remains unclear.

In this study, we investigated whether miRNAs in exosomes, especially before and after resistance to osimertinib, transmit relevant information between tumor cells, leading to changes in the sensitivity of patients. After collecting exosomes from the supernatant of osimertinib drug-resistant and drug-sensitive cells and comparing plasma exosomes of matched patients, a next-generation RNA-seq test was performed to identify key miRNAs. Later, we verified the role of key miRNAs in osimertinib resistance in larger population samples in an attempt to elucidate the mechanism of exo-miRNAs involved in osimertinib resistance. We hope to provide directions for the treatment of NSCLC patients with osimertinib resistance.

## Materials and methods

### Cell culture and establishment of resistant strains

Lung adenocarcinoma cell line H1975 cells (EGFR, L858R and T790M) were purchased from the Institute of Biochemistry and Cell Biology, Chinese Academy of Sciences (Shanghai, China). Osimertinib was obtained from Selleck Chemicals (Houston, TX, USA). The osimertinib-resistant cell line, H1975-OR, was induced and cultivated as previously reported [13]. All cells were cultured in RPMI 1640 medium containing 10% fetal bovine serum (FBS) and antibiotics (100 units/mL penicillin and 100 μg/mL streptomycin). All cells were incubated at 37 °C in 5% CO_2_ humidified air.

Cell proliferation was measured in three replicates using the MTT method as described previously [14]. A non-linear regression function was used to fit the dose–response curve in GraphPad Prism 5.0. After the IC_50_ values of the semi-inhibition rates of H1975 and H1975-OR cells were obtained, the drug resistance index was calculated. Both MTT and dimethyl sulfoxide (DMSO) were purchased from Sigma-Aldrich (St. Louis, MO, USA). RPMI 1640 medium, fetal bovine serum (FBS), penicillin, and streptomycin were purchased from Gibco Life Technologies (Grand Island, NY, USA).

### Patient selection and sample collection

A total of 67 NSCLC patients were included in this study and were observed in the Department of Respiratory and Critical Care Medicine at Jinling Hospital from December 2018 to October 2019. Three patients had a pair of blood samples collected each, before and after the appearance of resistance to osimertinib, to be compared with the H1975 cells samples. The samples of the remaining 64 patients were used for extended screening after identifying target miRNAs. Among these patients, 37 were resistant to gefitinib but had not yet developed osimertinib resistance, and 27 had Osimertinib resistance. In addition, 10 healthy people were recruited from the physical examination center of Jinling Hospital. All participants provided written informed consent. This study was approved by the ethics committee of Jinling Hospital (Ethical code: 2017NZGKJ-030).

### Isolation of exosomes

After reaching 80% confluency in a Petri dish, H1975 and H1975-OR cells were cultured in complete RPMI-1640 medium without added serum. The cell culture supernatant was collected 24 h later, and the exosomes were separated using differential ultracentrifugation as described previously [15]. To remove cell debris, the supernatant was centrifuged at 300×*g* for 5 min, at 3000×*g* for 10 min, and, lastly, at 10,000×*g* for 30 min. The supernatant was then passed through a 0.22-μm filter (Millipore, Burlington, MA, USA). The filtered supernatant was transferred to a clean ultracentrifuge tube and ultracentrifuged at 4 °C, 120,000×*g* for 70 min. After discarding the supernatant, the pellet was resuspended in an appropriate amount of sterile phosphate-buffered saline (1 × PBS). The extracted exosomes were used for immediate downstream experiments or stored in a -80 °C refrigerator.

Serum samples were extracted using a Total Exosome Isolation Kit (from serum) (4,478,360, Invitrogen, Carlsbad, CA, USA,). The required volume of clear serum was transferred to a new test tube and 0.2 volume of total exosome isolation (from serum) reagent was added. The serum and reagent were mixed, through eddy current or an up-and-down pipe motion, to make the solution turbid. After incubation at 4 °C for 30 min, the samples were centrifuged at 10,000×*g* for 10 min. The supernatant was aspirated and discarded. The exosomes, which were contained in the granules at the bottom of the test tube, were resuspended in half the volume of the initial serum volume of sterile PBS (1×).

### Transmission electron microscopy (TEM), size distribution analysis, and western blot

Exosome morphology was observed using TEM. The exosome suspension was mixed with an equal volume of 4% paraformaldehyde, and 10 μL of the mixture was placed on a clean copper grid (RT) at room temperature. Uranyl acetate staining was negative. The images were acquired by observation with a JEOL 1200EX TEMSCAN microscope. The exosomal suspensions were analyzed for particle size using dynamic light scattering (DLS) (Nanosizer ™ instrument, Malvern Instruments, Malvern, UK).

The extracted exosomes were resuspended in cell lysate (Beyotime, Nantong, China) supplemented with 1% PMSF. The protein concentration of exosomes was determined using a Pierce BCA protein detection kit (Thermo Fisher Scientific, Rockford, IL, USA). Sodium dodecyl sulfate polyacrylamide gel electrophoresis (SDS-PAGE) (12%) were prepared with 20 μg of protein on each sample. Anti-CD63, anti-TSG101, anti-β-actin, and anti-GAPDH were purchased from Abcam (Cambridge, UK). All antibodies used in western blot are diluted 1:1000.

### RNA-sequencing and raw data

The total RNA in the exosomes was extracted using TRIzol reagent (Invitrogen, Carlsbad, CA, USA) according to the manufacturer’s instructions. After performing the RNA extraction and quality control, the library was constructed and sequenced according to the literature. The raw data from small RNA-seq include linker sequences and sequencing low-quality sequences. In order to ensure the accuracy of the information analysis, the raw sequencing data were filtered to obtain clean data, and subsequent bioinformatics analysis was performed based on the clean data. The Qphred score (Qphred = 10log10 (e)) was used to represent the base quality value (Quality Score) to measure the quality of each base in the sequencing reads. The miRDeep2 software was used to analyze miRNA expression abundance. Cluster analysis and correlation analysis between samples (Pearson’s correlation coefficient) were performed on the miRNA family in each sample. All raw sequencing data were uploaded to the SRI database. [Submission ID: SUB7187450; BioProject ID: PRJNA615931] (http://www.ncbi.nlm.nih.gov/bioproject/615931).

### Target gene analysis

The analysis was performed using DESeq2 (no biological duplicate samples use DESeq or edgeR); miRNAs with [logFoldChage] > 1 and p value < 0.05 were selected as miRNAs with significant differences. A volcano map of the results of miRNA differential analysis and the clustered heat map of miRNA expression in the samples were drawn. TargetScan was used to calculate a weighted context + + score to predict the target genes of different known miRNAs. These target gene functions were classified through a database established using the Gene Ontology (GO) Consortium, and a GO enrichment analysis was performed. At the same time, the first 10 target genes of each sample were selected for Kyoto Encyclopedia of Genes and Genomes (KEGG) pathway enrichment analysis according to the parameters described in the literature to identify the downstream molecular metabolic pathways of these target genes.

### Quantitative reverse transcription PCR

The RNA extracted from the sera of patients was quantified and evaluated using NanoDrop® ND-2000 (Thermo Fisher Scientific, USA). We used the miRNA first-strand cDNA synthesis (tailing method) kit from Shanghai Sangon Biotech, using the method of Poly (A) tailing reaction and cDNA synthesis reaction simultaneously. After following the manufacturer’s instructions, all cDNA products of the miRNAs were obtained and real-time PCR experiments were performed. In the RT-PCR, the universal downstream primer Universal PCR Primer R and the endogenous reference U6 (Universal U6 Primer F) in the above kit were used uniformly. The upstream primers of several miRNAs are as follows: miR-184: 5'-catGGACGGAGAACtGAtAAGGGt-3 '; miR-3913-5p: 5'-acggTTTGGGACTGATCTTGATGTCT-3'; miR-4746-5p: 5'-CCGGTCCCAGGAGAACC-3'; miR-3614-5p: 5'-CCACTTGGATCTGAAGGCTGC-3'. RT-PCR was performed using an ABI 7500 real-time PCR system (Applied Biosystems, Foster City, CA, USA). The expression levels of all miRNAs were determined using the 2-ΔΔCT method: ΔCT (target) = CT (target) − CT (U6) [16]. All the above experiments were performed twice.

### Statistical analysis

The above data were mostly analyzed and mapped using the SPSS 22.0 system (SPSS, Inc. Chicago, IL, USA) and GraphPad Prism 5. The differences in the miRNA expression levels between the two groups were mainly determined using the Mann–Whitney rank-sum test for non-parametric data. The correlation between Exo-miR-184 and Exo-miR-3913-5p with clinicopathological features was determined using the χ2 test. ROC curves were also used to determine the diagnostic value. P < 0.05 values indicate a statistical difference.

## Results

### Isolation of exosomes from cell supernatant and serum

**Fig. 1:**
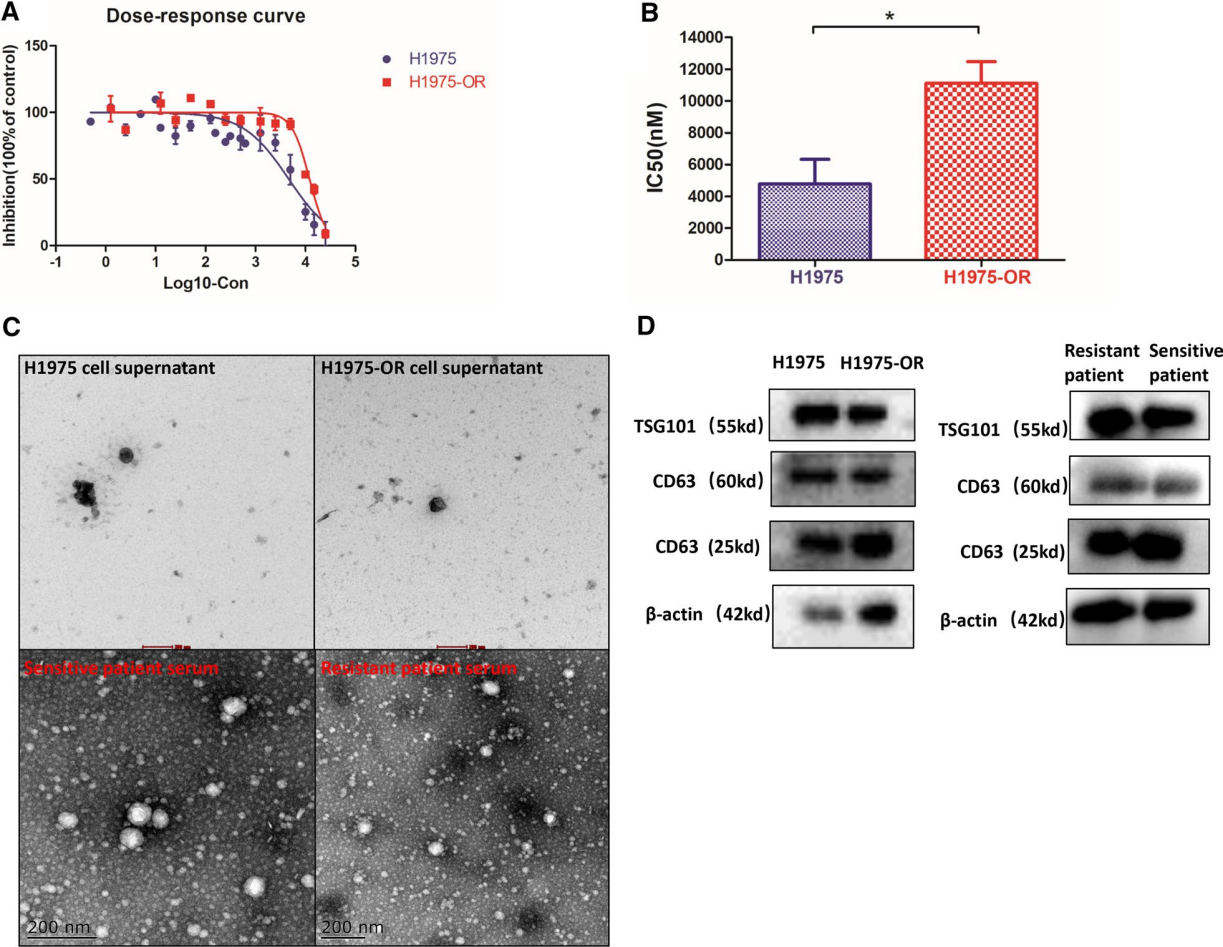
Isolation and identification of exosomes. **A** The drug-resistant strain, H1975-OR, was obtained by continuous exposure of H1975 to osimertinib for 6 months. After incubating H1975-OR and H1975 cells with different concentrations of osimertinib for 72 h, the cell viability was measured using the MTT method. **B** The average IC_50_ values in H1975 and H1975-OR were 4636 nM and 12,101 nM, respectively, and the difference was statistically significant (p = 0.0215). **C** The exosomes (30–150 nm) extracted from the H1975, H1975-OR cell supernatant, the osimertinib-sensitive and osimertinib-resistant patients serum were observed using electron microscopy. Scale = 200 nm. **D** Exosomes were validated by protein analysis in western blot

In the commonly used lung cancer cell lines, H1975 carries both EGFR-L858R drug-sensitive mutation and EGFR-T790M drug-resistant mutation. The drug-resistant strain H1975-OR was established by increasing the concentration of osimertinib. Six months later, the MTT method was used to determine the cell viability of the sensitive strain H1975 and the resistant strain H1975-OR (Fig. 1A). The average IC_50_ values of these two cells were 4636 nM and 12,101 nM, respectively (p = 0.0215) (Fig. 1B); RI was 2.61. Circular vesicle-like exosomes were obtained from different cell supernatants after ultracentrifugation. Blood samples were collected from patients with EGFR mutations before and after osimertinib resistance. The exosomes were extracted using the kit. Exosomes were also identified using TEM (Fig. 1C), western blotting (Fig. 1D), and DLS analysis (Additional file 1: Fig. S1A, B).

### Functional enrichment analysis of differentially expressed miRNA target genes in exosomes

**Fig. 2:**
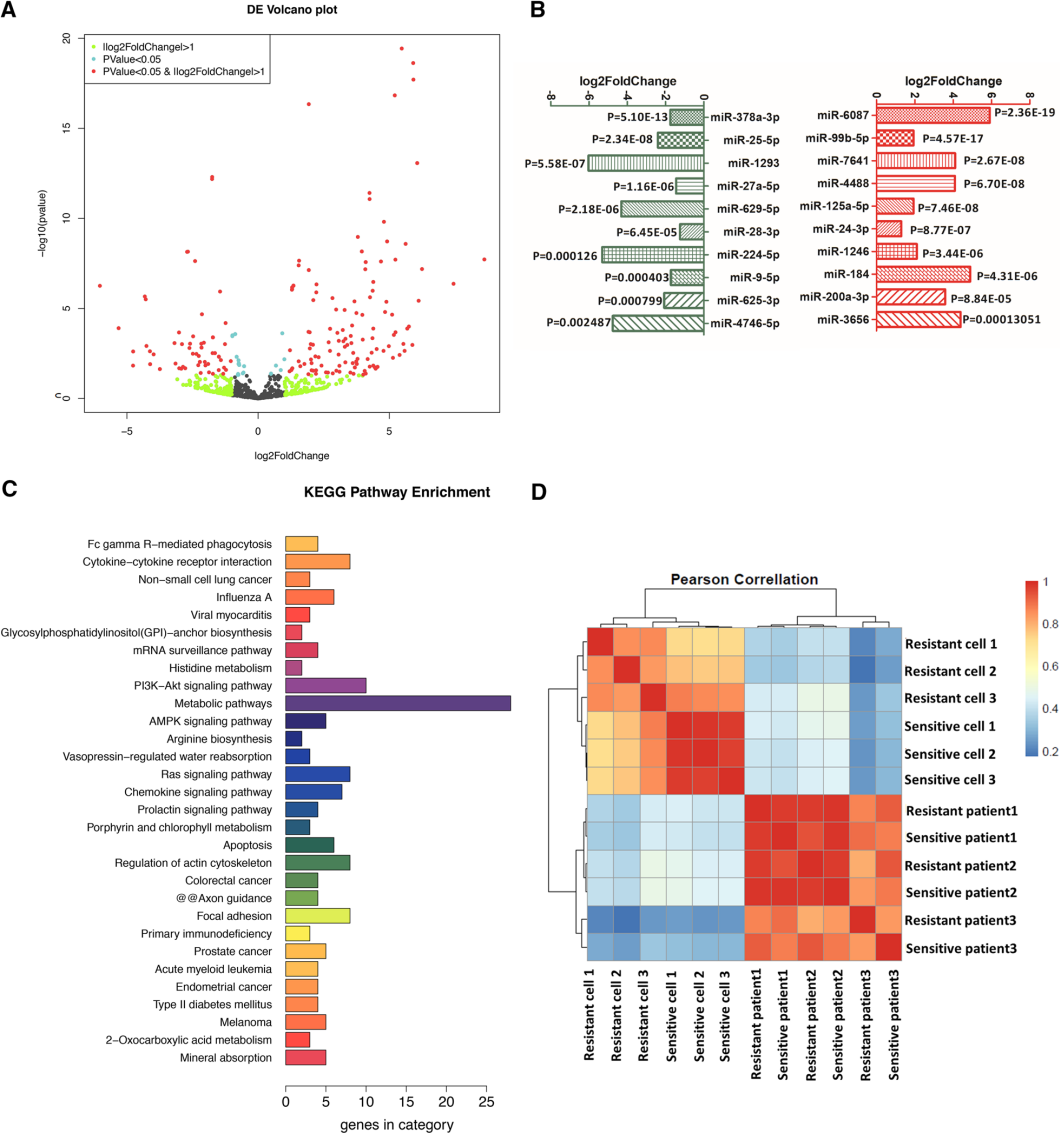
Cluster analysis of miRNAs and target genes. **A** Volcano map of differentially expressed miRNAs. Each point in the volcano map represents a gene, and the red dots are significantly different genes that satisfy both [logFC] > 1 and FDR < 0.05 thresholds. The blue dots are genes that only meet FDR < 0.05, and the green dots are genes that only meet [logFC] > 1. **B** H1975-OR top 10 up-regulated miRNAs (red) and top 10 down-regulated miRNAs (green) compared with H1975. **C** KEGG pathway enrichment analysis of target genes for differentially expressed miRNA (histogram). The ordinate represents the name of the pathway, and the abscissa is the number of genes involved in each pathway. **D** Calculate the Pearson coefficient of cell supernatant and patient serum.. The correlation coefficient uses Pearson’s correlation test (column, row), and the color of each square corresponds to the correlation coefficient value

The volcano map visually revealed the difference in miRNA expression of exosomes in the supernatant of H1975 and H1975-OR cells (Fig. 2A). Among them, the expression levels of exosome miR-6087, miR-99b-5p, and miR-7641 in the H1975-OR supernatant were significantly higher than those in the H1975 supernatant. Other exo-miRNAs, such as miR-378a-3p, miR-25-5p, and miR-1293, were significantly reduced (Fig. 2B). KEGG pathway enrichment was carried out for the top ten target genes of exosome miRNAs with differences between the two groups. The “metabolic pathway” involved the largest number of target genes, followed by the “PI3K-Akt signaling pathway,” “Ras signaling pathway,” “cytokine-cytokine receptor interaction,” “non-small cell lung cancer,” and other pathways (Fig. 2C). The pathway analysis of novel differentially expressed miRNA target genes focused on the MAPK signaling pathway (Additional file 1: Figure S1). These pathways are also members of the bypass pathways involved in drug resistance in EGFR-TKI therapy for NSCLC.

A 62-year-old male with NSCLC *(Patient 2, Treat3 & Control3)* (left lung adenocarcinoma, EGFR exon 19 deletion) was treated with gefitinib. One year later, he started oral AZD9291 due to new brain and bone metastasis. The cancer progressed after 19 months. Blood samples were collected from the patient at the start of osimertinib treatment and after presenting resistance. Similarly, blood samples were collected from a 53-year-old female patient (left lung adenocarcinoma stage IVB, EGFR exon 21 L858R mutation) *(Patient 1, Treat2 & Control2)* and a 58-year-old female patient (left lung adenocarcinoma stage IVB, EGFR exon 19 deletion) *(Patient 3, Treat4 & Control4)* before and after the appearance of resistance to osimertinib. Pearson's correlation coefficient (R2) was used to assess the correlation between exo-miRNAs from the cell supernatant and serum of patients, and the correlation heat map matrix was drawn (Fig. 2D).

### Elevated miR-184 and miR-3913-5p in exosomes after osimertinib resistance

**Fig. 3:**
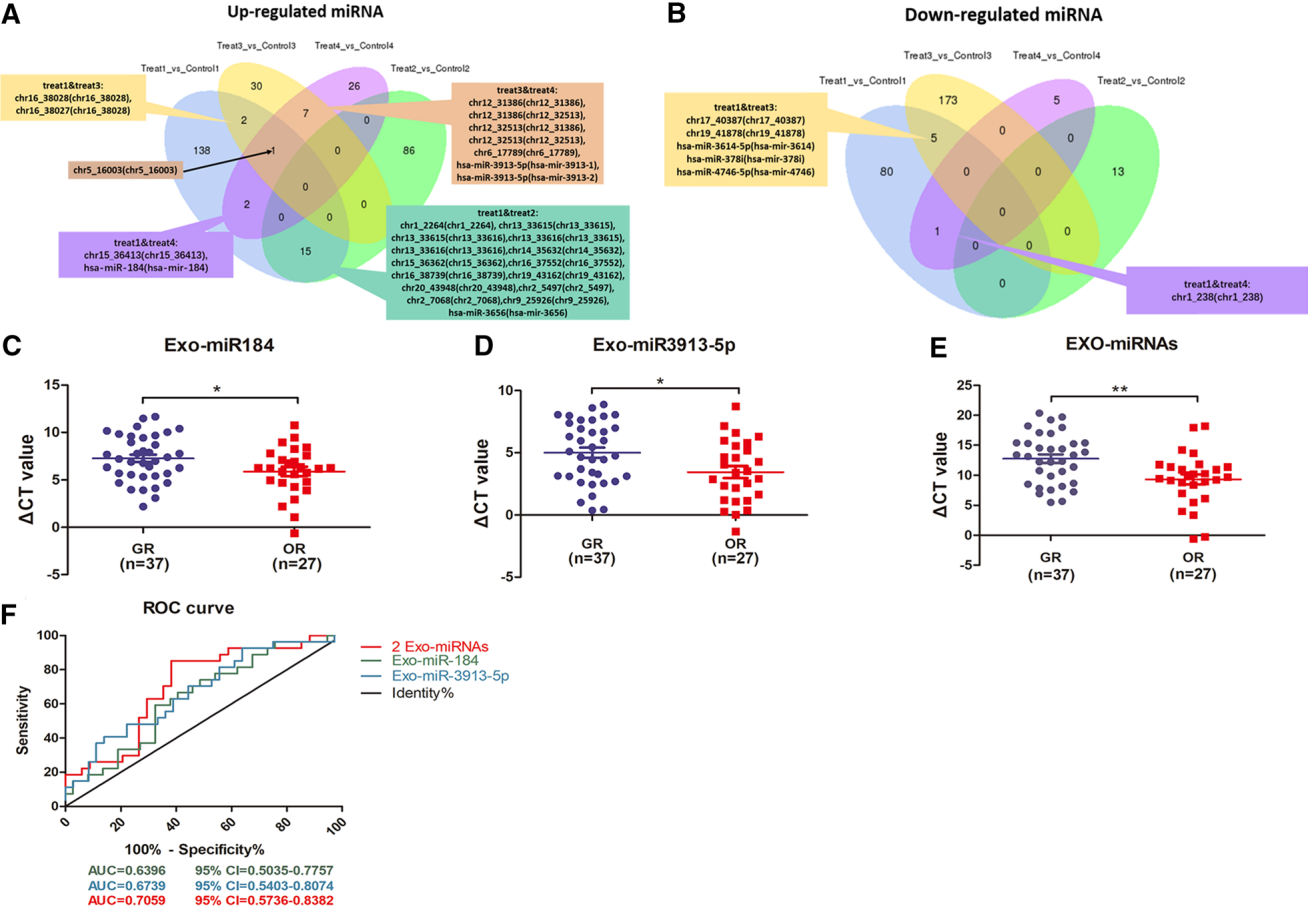
MiR-184 and miR-3913-5p elevated expression in exosomes after osimertinib resistance. **A** Overlaid analysis of Wayne maps of exosomal miRNAs up-regulated and **B** down-regulated exosomal miRNAs in the drug-resistant group compared to that in the sensitive group. **C** q-PCR exosome miR-184 expression levels in patients with osimertinib-resistant NSCLC (OR, n = 27) and patients without resistance (GR, n = 37), * p < 0.05. **D** Serum exosomal miR-3913-5p expression levels in patients with osimertinib-resistant NSCLC (OR, n = 27) and osimertinib-sensitive patients (GR, n = 36), * p < 0.05. **E** Changes in the expression levels of the two exosomal miRNAs before and after resistance to osimertinib, **p < 0.01. **F** ROC curves for miR-184 (green), miR-3913-5p (blue), and the two miRNAs (red), respectively

We performed an overlapping analysis of the differential miRNAs obtained by sequencing exosomes in the cultured cells supernatant (treat1) and the blood samples of three patients (treat2-4) using a Wayne map (Fig. 3A, B). Among the exo-miRNAs upregulated in the osimertinib-resistant group compared to those in the sensitive group, no common differential miRNAs were found to overlap in the four groups **(**Fig. 3A). However, in a pairwise comparison, it was found that miR-184 overlapped in treat1 and treat4, miR-3913-5p appeared both in treat3 and treat4, and miR-3656 co-occurred in treat1 and treat2 (Fig. 3A). These three miRNAs were further studied. Similarly, miR-3614-5p, miR-4746-5p, and miR-378i were chosen as the target miRNAs in the down-regulated miRNA group (Fig. 3B).

In order to verify several significantly up-regulated exo-miRNAs in the RNA-seq results, we decided to expand the sample size for experiments. Serum samples from 37 NSCLC patients with gefitinib resistance and 27 patients with osimertinib resistance were collected as controls (GR) and experimental groups (OR). Using QPCR, we verified that the exosome miR-184 in the serum of drug-resistant patients was significantly high (p = 0.0325) (Fig. 3C), and exosome miR-3913-5p was significantly increased in the osimertinib-resistant group (p = 0.0169) (Fig. 3D), which was consistent with the previous sequencing results.

### Increased miRNAs suggested that NSCLC patients were resistant to osimertinib

Combining these two exo-miRNAs, it was found that these two miRNAs in the serum exosomes of NSCLC patients were significantly upregulated after osimertinib resistance (p = 0.0092) (Fig. 3E, F). The relationship between exo-miRNA expression levels and the clinicopathological characteristics of the patients (e.g., age, sex, smoking history, TNM stage, primary tumor size, platelet count (PLT), lactate dehydrogenase (LDH), carcinoembryonic antigen (CEA), and distant metastasis) is summarized in Tables 1 and 2. Exosome miR-184 was correlated with LDH levels (p = 0.018) (Table 1); exosome miR-3913-5p was associated with TNM stage (p = 0.045), PLT (p = 0.024), tumor marker CEA (p = 0.045), distant metastases (p = 0.049), and, especially, bone metastasis (p = 0.03) (Table 2). However, there was no significant correlation between miRNA expression levels of the two exosomes and age, sex, smoking status, ECOG score, and primary tumor size.

**Table 1:** Correlation between serum exosomal miR-184 expression levels in patients with NSCLC and their clinicopathological characteristics. (* p < *0.05)*

Clinicopathological parameters	Number of patients(%)	Exosomal miR-184		
		Low	High	*P*
Age(years), Median				0.777
< 60	33	15	18	
≥ 60	31	13	18	
Gender				0.899
Male	28	12	16	
Female	36	16	20	
Smoking history				0.353
Never smoking	49	23	26	
Now/once smoking	15	5	10	
ECOG Score				0.226
0–1	53	25	28	
≥ 2	11	3	8	
Primary tumor				0.705
≤ 4 cm	19	9	10	
> 4 cm	45	19	26	
TNM				0.317
I–IVA	25	9	16	
IVB	39	19	20	
PLT				0.411
Low(≤ 146)	13	7	6	
High(> 146)	51	21	30	
LDH				0.018*
Low(≤ 199)	29	8	21	
High(> 199)	35	20	15	
CEA				0.129
Low(≤ 8.605)	25	8	17	
High(> 8.605)	39	20	19	
Distant metastasis				0.164
No	17	5	12	
Yes	47	23	24	
Contralateral lung metastasis				0.069
No	24	7	17	
Yes	40	21	19	
Pleura metastasis				0.825
No	33	14	19	
Yes	31	14	17	
Bone metastasis				
No	23	9	14	
Yes	41	19	22	
Brain metastasis				
No	36	14	22	
Yes	28	14	14

**Table 2:** Correlation between serum exosomal miR-3913-5p expression levels in patients with NSCLC and their clinicopathological characteristics. (* p < *0.05)*

Clinicopathological parameters		Number of patients(%)		Exosomal miR-3913-5p				
				Low		High		*P*
Age(years), Median								0.714
< 60		32		25		7		
≥ 60		31		23		8		
Gender								0.427
Male		28		20		8		
Female		35		28		7		
Smoking history								0.321
Never smoking		48		38		10		
Now/once smoking		15		10		5		
ECOG Score								0.064
0–1		52		42		10		
≥ 2		11		6		5		
Primary tumor								0.64
≤ 4 cm		18		13		5		
> 4 cm		45		35		10		
TNM								0.045*
I–IVA		24		15		9		
IVB		39		33		6		
PLT								0.024*
Low(≤ 146)		13		13		0		
High(> 146)		50		35		15		
LDH								0.427
Low(≤ 199)		28		20		8		
High(> 199)		35		28		7		
CEA								0.045*
Low(≤ 8.605)		24		15		9		
High(> 8.605)		39		33		6		
Distant metastasis								0.049*
No		17		10		7		
Yes		46		38		8		
Contralateral lung metastasis								0.434
No		24		17		7		
Yes		39		31		8		
Pleura metastasis								0.414
No		32		23		9		
Yes		31		25		6		
Bone metastasis								0.03*
No		23		14		9		
Yes		40		34		6		
Brain metastasis								0.112
No		35		24		11		
Yes		28		24		4		

### Exosomal-derived miRNAs predict osimertinib resistance in patients with EGFR exon21 L858R mutation.

**Fig. 4:**
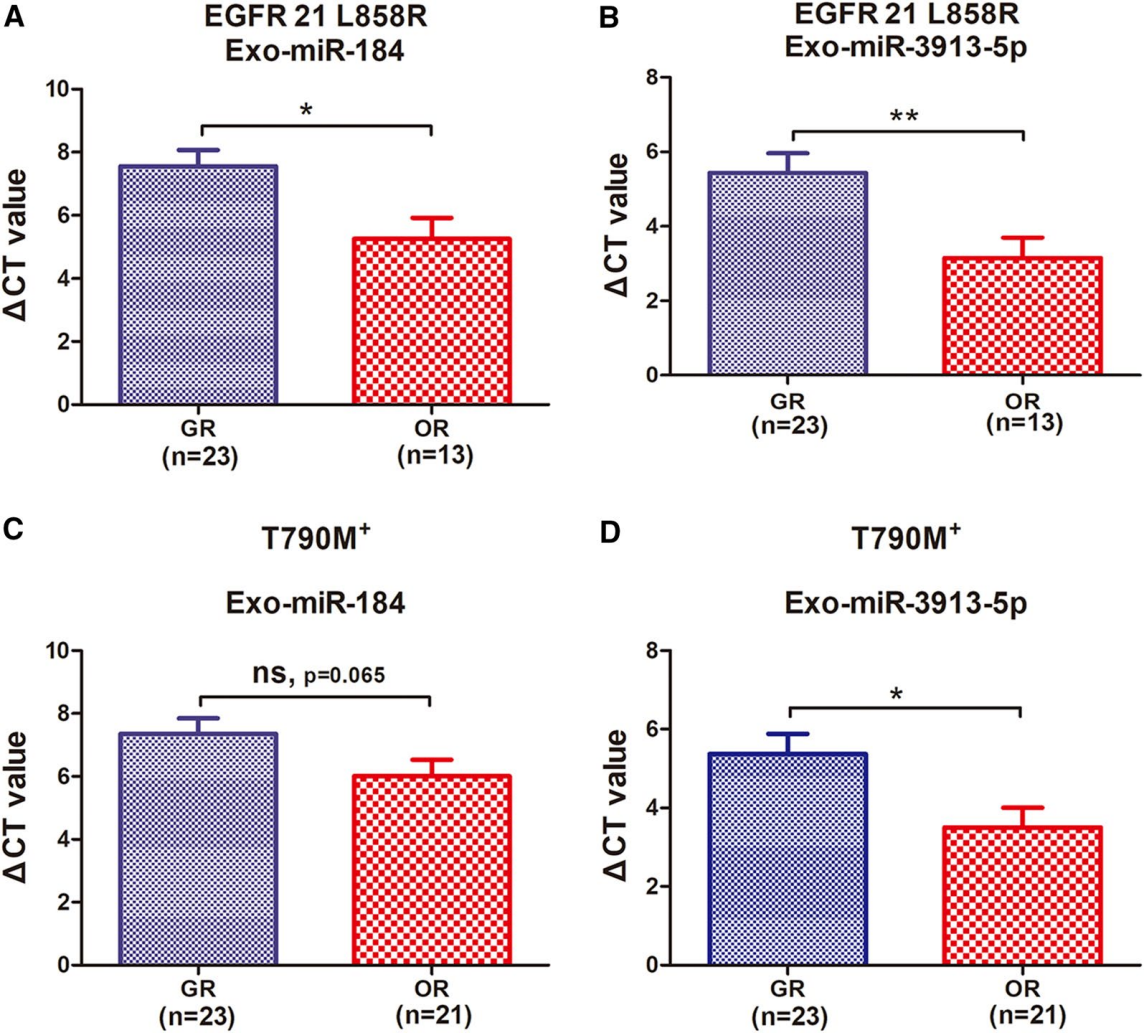
Osimertinib resistance induced by exosomal miRNAs targeting EGFR 21 mutations and T790M + patients. **A** q-PCR analysis of serum exosomal miR-184 and **B** miR-3913-5p expression levels in patients with drug resistance and sensitivity, in all patients with EGFR 19 exon deletion. **C** q-PCR analysis of serum exosomal miR-184 and **D** miR-3913-5p expression levels in patients with resistance and sensitivity in all patients with EGFR 19 exon L858R mutation

Of the 64 NSCLC patients, 28 possessed EGFR exon 19 deletions and 36 had mutations in the EGFR exon 21. In all patients with exon 19 deletion, there was no significant difference in the expression levels of serum exosomal miR-184 (p = 0.776) and miR-3913-5p (p = 0.631) between the groups before and after drug resistance **(**Additional file 1: Fig. S4A, B**)**. However, in all patients with mutations in L858R in the EGFR exon 21, the serum expression level of exo-miR-184 was significantly elevated in osimertinib-resistant patients (p = 0.0104) (Fig. 4A). Exo-miR-3913-5p (p = 0.0085) was also significantly altered in patients with osimertinib resistance (Fig. 4B). ROC curve analysis showed that the AUC of exo-miR-184 was 0.736, while the AUC of exo-miR-3913-5p was 0.759 **(**Additional file 1: Fig. S4C, D**)**, indicating that these two miRNAs play important roles in osimertinib resistance and were mainly involved in drug resistance in patients with EGFR exon 21 L858R. Because the sensitive group (GR) was selected for gefitinib-resistant patients, the criteria for clinical resistance in these patients were enlarged lesions or new organ metastases. After re-sequencing, most patients (n = 44) showed T790M-positive mutations in EGFR-TKI resistance, but a few patients (n = 20) were not T790M-positive **(**Additional file 1: Fig. S4**)**. In T790M + osimertinib-resistant patients, the expression level of exo-miR-3913-5p was significantly increased (p = 0.013) (Fig. 4D), and the exo-miR-184 expression level in the resistant group was also higher than that in the sensitive group, but the difference was not statistically significant (p = 0.065) (Fig. 4C).

## Discussion

Osimertinib (AZD9291) is the first third-generation EGFR-TKI to be approved by the FDA and EMA for the treatment of NSCLC [5]. It is selective for EGFR-TKI sensitization and T790M resistance mutations and has less effect on WT-EGFR [17]. However, similar to the findings observed after the use of other EGFR-TKIs, patients still inevitably develop resistance after receiving osimertinib, regardless of its first- or second-line use, which greatly limits the long-term clinical benefits of this targeted drug [18]. The mechanism of osimertinib resistance depends on the high tumor heterogeneity of NSCLC, which is divided into EGFR-dependent and EGFR-independent aspects [19]. Moreover, previous studies have shown that the resistance mechanism after first- or second-line use of osimertinib varies with clonal evolution [20]. Our study focused on patients with advanced NSCLC, who were treated with non-first-line osimertinib. Patients enrolled in the sensitive group (GR) were those who were sensitive to osimertinib after gefitinib resistance, while patients in the resistant group (OR) were those who had developed clinical resistance to osimertinib. This ensured the homogeneity of these two sample groups.

The most common EGFR-dependent drug-resistance mechanism is C797S mutation occurring on exon 20, which has previously been reported to account for 10–26% of all cases using osimertinib as second-line therapy [21]. In addition to C797S-adjacent G796 mutation, there are multiple mutation sites possibly involved, which include L792, L718, G719, G724, and EGF overexpression [22]. However, in our study, of the 27 patients with osimertinib resistance, only 3 (11.11%) had a C797S mutation that was confirmed based on molecular testing. Of course, there were also several patients who did not undergo genetic testing again for financial reasons.

The most important EGFR-independent mechanisms of drug resistance are activation of bypass signals, abnormalities in downstream pathways, and histological transformation. The most common activated bypass pathways are MET amplification [23] and HER2 amplification [24]. The abnormality of the RAS-MAPK pathway is also an important mechanism of osimertinib resistance. Ortiz-Cuaran et al. [25] confirmed that when acquired resistance to second-line osimertinib was developed, re-biopsy of the tumor revealed a KRAS G12S mutation. Kim et al. [26] reported a case of MAPK1 mRNA overexpression in a patient who received second-line treatment with osimertinib in advanced stages. This is consistent with our exosome sequencing results. As shown in Fig. 2C, the most obvious pathway enriched by the known differential exo-miRNA target gene in the supernatants of drug-resistant (H1975-OR) and sensitive (H1975) strains was the Ras signaling pathway. As shown in Additional file 1: Figure S2, the MAPK signaling pathway is the most abundant among the novel differential miRNA target genes with the largest number of genes involved. The above results indicated that the participation of exo-miRNAs in osimertinib resistance was also related to abnormalities in the RAS-MAPK pathway. In addition, the PI3K pathway is involved in bypass activation. It is currently believed that PIK3CA mutation or amplification and PTEN deletion can lead to activation of the PI3K pathway [26]. In our study, the PI3K-Akt signaling pathway was observed in KEGG enrichment analysis. Exo-miRNAs may convey osimertinib resistance information to affect the activation of the PI3K pathway. Osimertinib resistance is also related to changes in cell cycle genes, including cyclin D1, cyclin D2, cyclin E1, cyclin-dependent kinase (CDK) 4, and CDK6 [27]. In the sequencing results, exosome miR-6087 was significantly increased in the drug-resistant group. Its target gene was CCND1 (Additional file 1: Table S1), which encodes the cyclin D1 protein. This again demonstrated that exosomes participated in osimertinib resistance by having an impact on bypass activation.

As mentioned previously, exosomes can contain a large number of proteins, nucleic acids, and lipids, which transmit information between cells [28]. Tumor-derived exosomes can be detected in the blood and body fluids of patients. It has been demonstrated that exosomes can affect the therapeutic response and induce drug resistance in tumor cells [29]. Recent research has suggested that drug-resistant cells transmit drug resistance information to drug-sensitive cells through extracellular vesicles [30]. The miRNAs, which are short non-coding RNA, have been thoroughly studied in oncology [31]. When miRNAs are loaded into exosomes, they can be protected from degradation by RNases [32]. An interesting finding was the discovery that exo-miRNAs could assist in the diagnosis of NSCLC. We compared the levels of exosome miR-184, miR-3913-5p, miR-3614-5p, and miR-4746-5p in the serum of NSCLC patients and healthy individuals, and found significant differences between the two. The area under the curve (AUC) of miR-184 was 0.803 (95% confidence interval: 0.701–0.905), greater than 0.75, indicating that miR-184 of serum exosomes of lung cancer patients could be used as a biomarker for the diagnosis of NSCLC (Additional file 1: Figure S5).

In recent years, many papers have been published on the relationship between exosome derived miRNAs and drug resistance in the treatment of NSCLC [33, 34]. However, few studies have discussed the relationship between the resistance to the third-generation EGFR-TKI osimertinib and exosomes. In our study, we observed exosomes extracted from the cultured H1975 and H1975-OR cell supernatant and the serum of three patients. Changes in the expression levels of exo-miRNAs after osimertinib resistance were captured. We identified, for the first time, that the increased levels of miR-184 and miR-3913-5p can predict resistance to osimertinib in NSCLC patients treated with this drug. In the clinicopathological features, LDH has been proven to be closely related to clinical prognosis in a variety of malignant tumors [35]. NSCLC patients with higher LDH levels have a worse prognosis and shorter survival than those with lower levels [36]. Although the exosome miR-184 found in our study was related to LDH level (p = 0.018) because of the time limit of this experiment, we could not continue to follow up to obtain survival data; therefore, whether exosome miR-184 could be a prognostic indicator remains unclear. CEA is the most common biomarker of lung adenocarcinoma [37]. It has been confirmed that an increase in CEA levels during TKI treatment for EGFR mutation patients may be a more sensitive predictor of an explosive progression in lung adenocarcinoma [38]. PLT is often associated with the platelet-to-lymphocyte ratio (PLR) [39]. Studies have suggested that the preoperative PLT-PLR score could be of significance in predicting the prognosis of patients with surgically resected NSCLC [40]. In this study, we found that exo-miR-3913-5p was related to TNM stage (p = 0.045), PLT (p = 0.024), CEA (p = 0.045), distant metastasis (p = 0.049), and bone metastasis (p = 0.03). Moreover, the AUC in the receiver operating characteristic curve was greater than 0.75, which further suggested that the exosome miR-3913-5p expression level was associated with advanced progression of lung adenocarcinoma in patients with EGFR mutations during TKI use.

Venn diagram analysis demonstrated that miR-184 expression level alteration was overlapped in Treat1 (H1975 lines cells) and Treat4 (A patient with EGFR exon 19 del). MiR-3913-5p expression level alteration was found to be overlapped in Treat3 and Treat4, and both patients had EGFR exon 19 deletions. However, we found in these subgroup analyses that the expression levels of both miRNAs were significantly altered in patients with EGFR exon 21 L858R point mutations. Previous studies have found that exon 19 deletion mutations (55.0%) have a higher rate of T790M resistance mutations than exon 21 L858R point mutations (37.3%) [41]. Recent studies have shown that the hazard ratio of survival benefit for Asian and L858R mutant populations is close to 1.00 in all people receiving osimertinib treatment [6]. Our study found that the expression levels of these two exo-miRNAs changed significantly in the L858R mutant population; however, this phenomenon was not observed in the population with exon 19 deletion. Thus, these two exosome-derived miRNAs might be more useful to predict resistance to osimertinib in patients with EGFR exon 21 L858R point mutations than in other populations. Previous studies have reported that nearly half of the patients lost the T790M mutation at the time of progression to osimertinib [42], and this loss may be related to the early resistance to osimertinib. The loss of T790M mutation is not conducive to prognosis [43]. Plasma T790M levels may predict acquired resistance [44]. However, considering that osimertinib is selective for EGFR sensitivity and T790M mutation, scholars believe that the emergence of a T790M mutation under osimertinib treatment is not a drug-resistance mechanism [19]. However, in this study, we found that exosomal miR-3913-5p expression levels were significantly altered in T790M-positive patients, indicating that this exo-miRNA may be involved in the drug-resistance mechanism in such patients. Exosomal miR-184 and miR-3913-5p are likely to be important molecules for the transmission of osimertinib resistance.

Our study had some limitations. The number of patients validated in this study was 64, and the number of collected specimens was not ideal. Only patient serum samples were used to extract exosomes, and no further humoral exosomes were used for verification. The clinical characteristics of the patients were collected, but no survival analysis was performed owing to the limitation of follow-up time. The predicted target genes and pathways will be verified in our subsequent experiments.

Our experiment demonstrated that exosome-derived miRNAs may be involved in the mechanisms of resistance to the third-generation EGFR-TKI osimertinib, especially affecting bypass pathway activation. Therefore, an increase in the expression levels of exosome-derived miR-184 and miR-3913-5p in the peripheral blood of NSCLC patients receiving osimertinib treatment may indicate the development of drug resistance. We hope that this study helps to expand the application of liquid biopsy technology in the field of clinical drug resistance in lung cancer. This may not only favorably guide clinical treatment but also provide direction for the development of a new generation of targeted drugs.

## Supplementary Information

**Additional file 1: Figure S1:** (A) and (B) particle size analysis of exosomes in the cell supernatant and plasma of patients, respectively. **Figure S2:** Pathway enrichment analysis of differentially expressed miRNA target genes. **Figure S3.** Differentially expressed exosome-derived miRNAs before and after osimertinib resistance. **Figure S4.** Exosome-derived miRNAs related to osimertinib resistance in patients with T790M mutation. **Figure S5.** Diagnostic value of exosomal miRNAs for NSCLC. **Table S1.** Exosomal miRNAs-induced osimertinib resistance by the activation of bypass pathways.

## Data Availability

The datasets used and/or analyzed during this study are available from the corresponding author upon reasonable request.

## Abbreviations

NSCLC: Non-small cell lung cancer
TKI: Tyrosine kinase inhibitor
EGFR: Epidermal growth factor receptor
BRAF: Serine/threonine protein kinase b-Raf
T790M: Thr790Met
DMSO: Dimethyl sulfoxide
FBS: Fetal bovine serum
PLT: Platelet count
LDH: Lactate dehydrogenase
CEA: Carcinoembryonic antigen
