# Supplementary material for: Elevated exosome-derived miRNAs predict osimertinib resistance in non-small cell lung cancer
Source: Cancer Cell Int. 2021 Aug 14;21:428. doi: 10.1186/s12935-021-02075-8 (PMC8364701; doi:10.1186/s12935-021-02075-8)
Supplement: Supplementary file 1 — Additional file 1: Figure S1: (A) and (B) particle size analysis of exosomes in the cell supernatant and plasma of patients, respectively. Figure S2: Pathway enrichment analysis of differentially expressed miRNA target genes. Figure S3. Differentially expressed exosome-derived miRNAs before and after osimertinib resistance. Figure S4. Exosome-derived miRNAs related to osimertinib resistance in patients with T790M mutation. Figure S5. Diagnostic value of exosomal miRNAs for NSCLC. Table S1. Exosomal miRNAs-induced osimertinib resistance by the activation of bypass pathways. [file 12935_2021_2075_MOESM1_ESM.docx]

Additional file

Elevated exosome-derived miRNAs predict osimertinib resistance in non-small cell lung cancer

Additional Figures

**Figure S1**

**
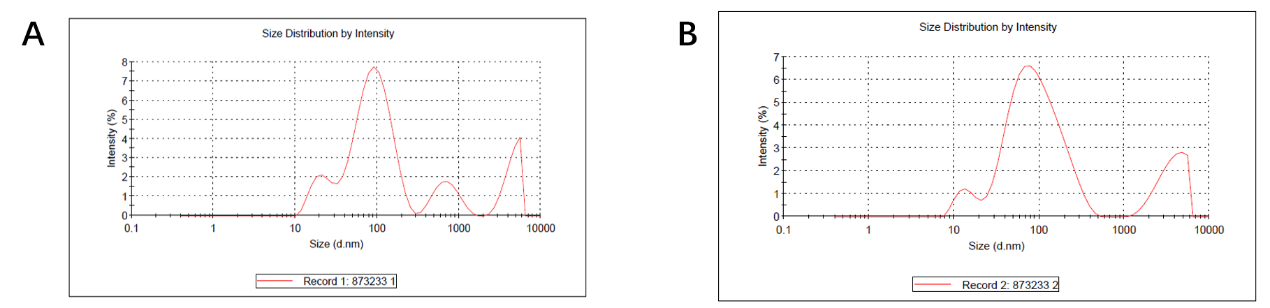
**

**Figure S1: (A) and (B) particle size analysis of exosomes in the cell supernatant and plasma of patients, respectively.**

**Figure S2**


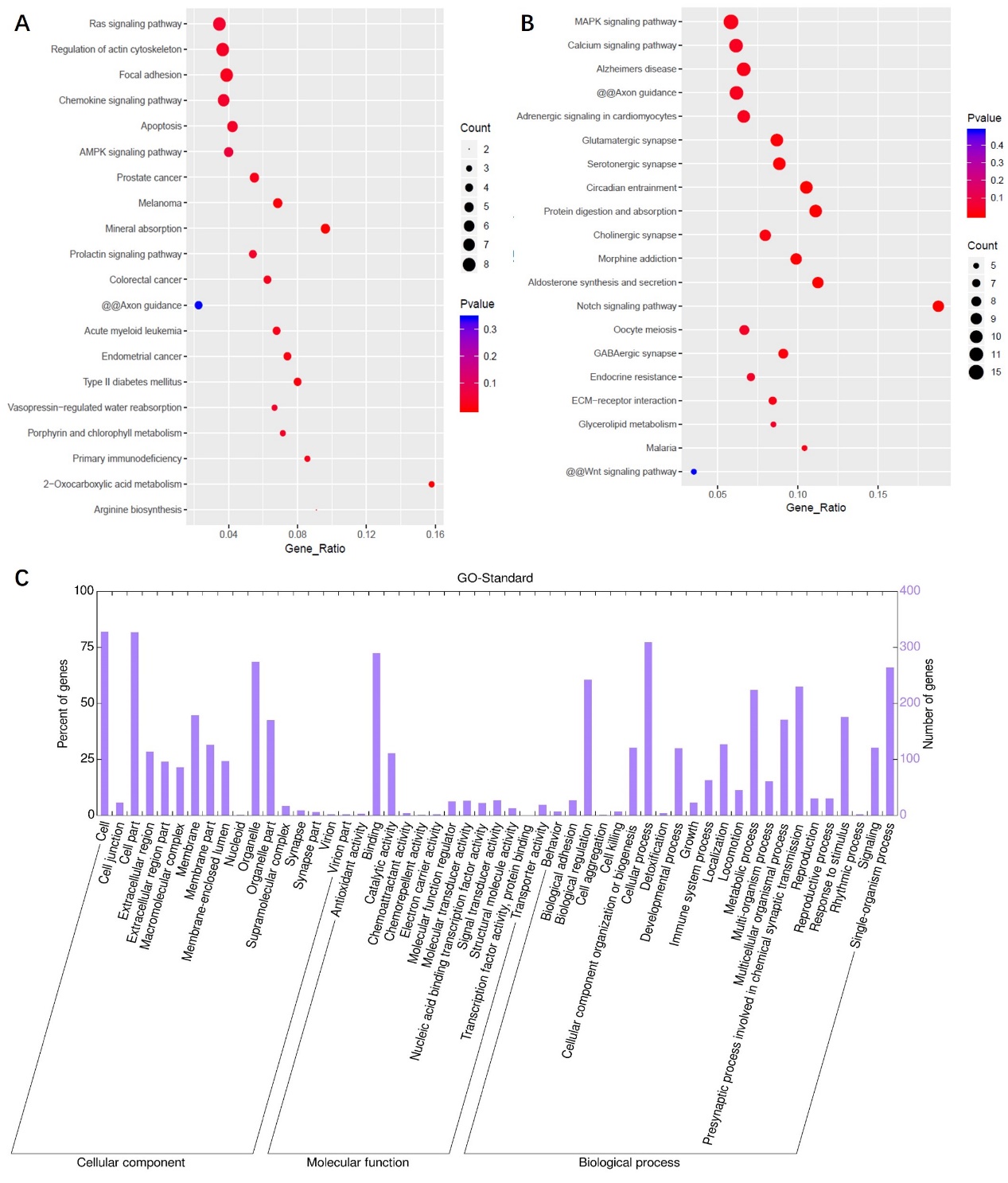


**Figure S2: Pathway enrichment analysis of differentially expressed miRNA target genes.**

(A) KEGG Pathway enrichment analysis of differentially expressed miRNA target genes (dot plot). The ordinate indicates the name of the pathway and the abscissa indicates the number of genes enriched in this pathway. The dot size indicates the size of the enrichment factor. The larger the point, the larger the enrichment factor. The larger the enrichment factor, the more prominent the annotation. A dot represents a pathway and different colors indicate the size of the P value. The smaller the P value, the more significant the enrichment.

(B) KEGG Pathway enrichment analysis of NOVEL differentially expressed miRNA target genes (dot plot).

(C) GO enrichment analysis of target genes for differentially expressed miRNAs. These target genes are divided into three categories: biological process, molecular function, and cellular component. List of the number of genes (right) and proportions (left) involved in each pathway.

**Figure S3**


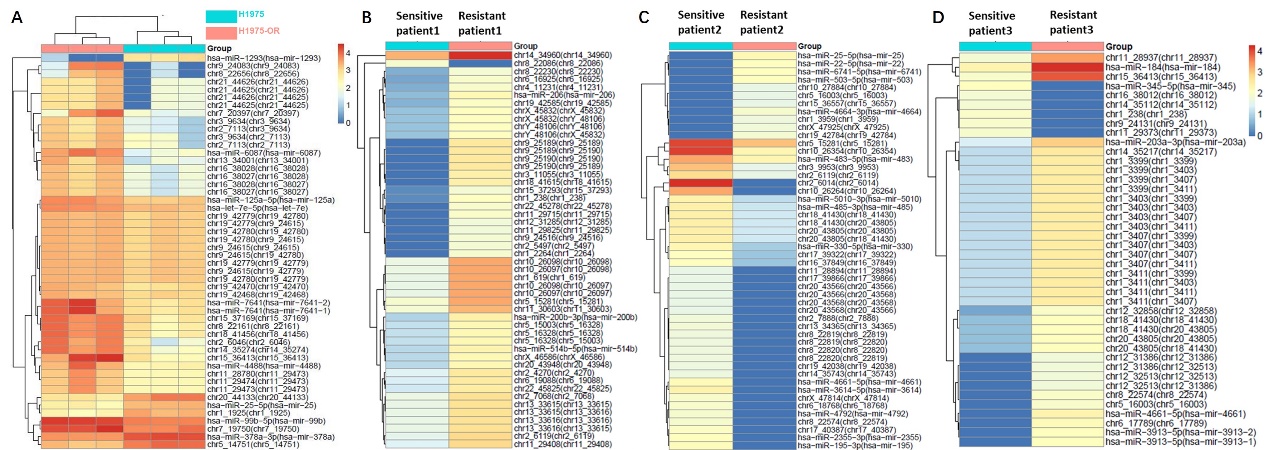


**Figure S3: Differentially expressed exosome-derived miRNAs before and after osimertinib resistance.**

(A) Hierarchical cluster analysis of differentially expressed miRNAs. The vertical column represents the sample, each row represents a miRNA, the right side is marked with the miRNA name, and the color represents the expression levels (normalized read count is taken as log10), and the red to blue represents a gradually decreasing expression level. (B) Hierarchical cluster analysis of differentially expressed miRNAs in serum exosomes of 3 patients. Patient 1: Female, 53 years old, left lung adenocarcinoma stage IVB, EGFR 21 L858R mutation (Treat2 & Control2); (C) Patient 2: Male, 62 years old, left lung adenocarcinoma stage IVB, EGFR 19 deletion (Treat3 & Control3); (D) Patient 3: Female, 58 years old, left lung adenocarcinoma stage IVB, EGFR 19 deletion (Treat4 & Control4).

**Figure S4**


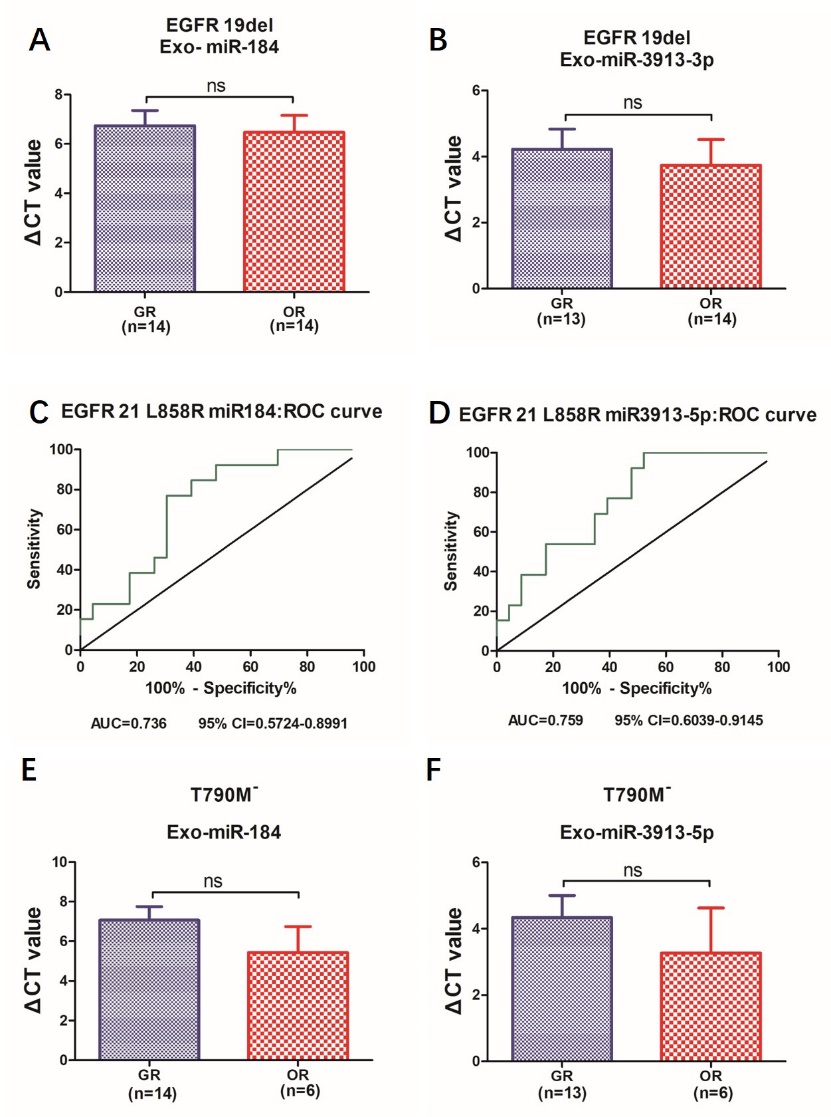


**Figure S4: Exosome-derived miRNAs related to osimertinib resistance in patients with T790M mutation.**

(A) q-PCR analysis of serum exosome-derived miR-184 and (B) miR-3913-5p expression levels in patients with drug resistance and sensitivity, in all patients with EGFR 19 exon deletion.

(C) (D) ROC curve of the two miRNAs in serum exosomes of patients with EGFR 21 exon L858R mutation.

(E) q-PCR analysis of miR-184 and (F) miR-3913-5p expression levels in serum exosomes of drug-resistant and sensitive groups, ns: p ≥0.05.

**Figure S5**


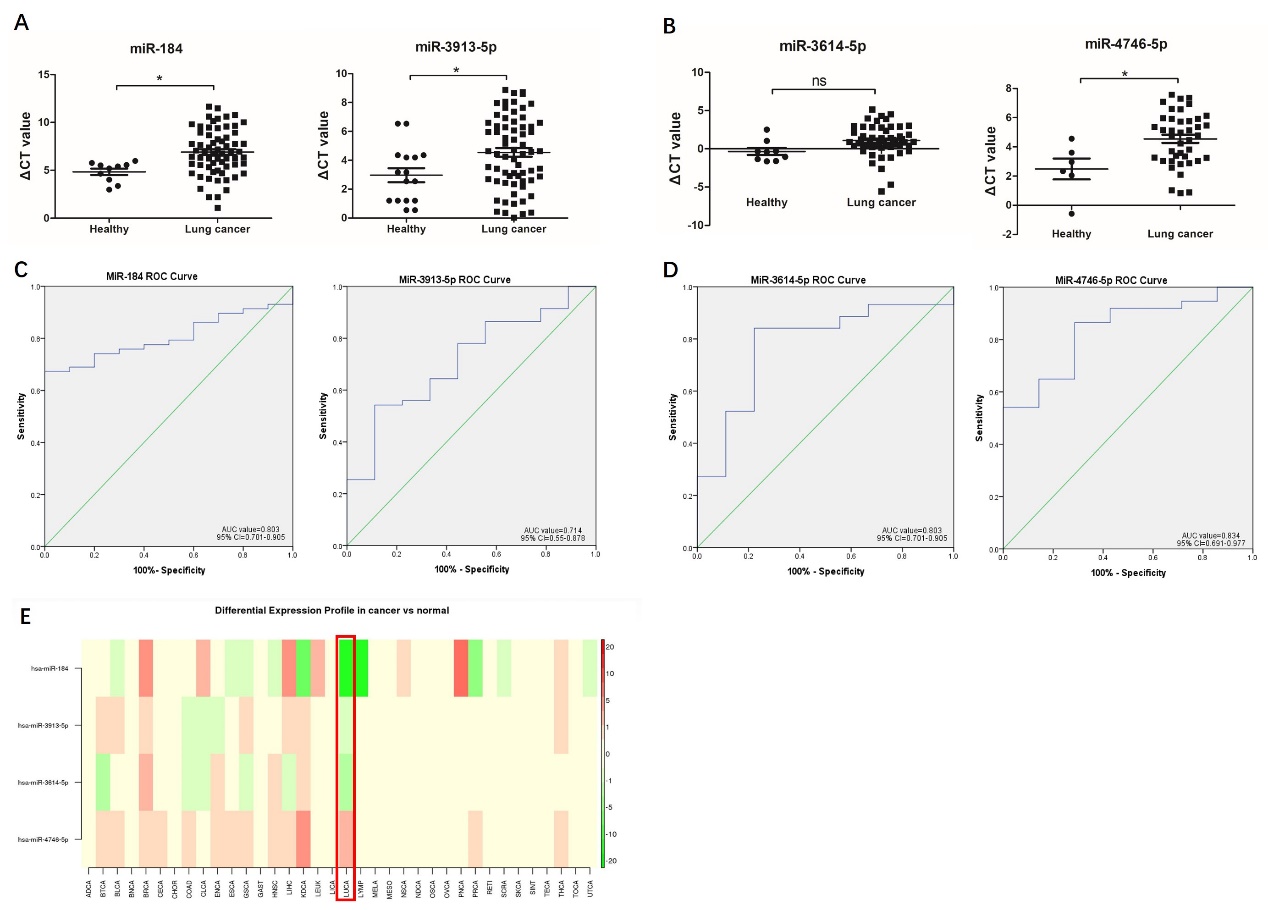


**Figure S5: Diagnostic value of exosomal miRNAs for NSCLC.**

1. q-PCR was used to determine the expression levels of exosomal miR-184 and miR-3913-5p in lung cancer patients (n = 64) and healthy controls (n = 10), * p <0.05.
2. q-PCR was used to determine the expression levels of exosomal miR-3614-5p and miR-4746-5p in lung cancer patients (n = 64) and healthy controls (n = 10), * p <0.05.
3. (D) The four exosomal miRNAs used as diagnostic criteria for NSCLC and their ROC curves.

(E) miRBase database (http://www.mirbase.org/) information for miR-184, miR-3913-5p, miR-4746-5p, and miR-3614-5p expression in different tumors compared with healthy controls. Red is up-regulated and green is down-regulated. The darker the color, the more obvious the expression gap.

Additional Table

**Table S1: Exosomal miRNAs-induced osimertinib resistance by the activation of bypass pathways.**

| ***Term*** | ***GeneName*** | ***miRNA*** | ***log2FoldChange*** | ***stat*** |
| --- | --- | --- | --- | --- |
| **Ras signaling pathway** | PDGFC | hsa-let-7d-3p(hsa-let-7d) | 0.020946 | up |
|  | NGF | hsa-let-7e-5p(hsa-let-7e) | 4.10E-08 | up |
|  | FGF7 | hsa-miR-30e-5p(hsa-mir-30e) | 0.012066 | up |
|  | FGF7 | hsa-miR-30c-5p(hsa-mir-30c-2) | 0.026564 | up |
|  | FGF7 | hsa-miR-30c-5p(hsa-mir-30c-1) | 0.025462 | up |
|  | PIK3CD | hsa-miR-7704(hsa-mir-7704) | 0.000227 | up |
|  | GNGT2 | hsa-miR-1246(hsa-mir-1246) | 3.44E-06 | up |
| **Ras signaling pathway** | BAD | hsa-miR-1292-5p(hsa-mir-1292) | 0.009353 | down |
|  | RASA3 | hsa-miR-95-3p(hsa-mir-95) | 0.003583 | down |
|  | PLA2G2A | hsa-miR-9-5p(hsa-mir-9-3) | 0.000403 | down |
|  | PLA2G2A | hsa-miR-9-5p(hsa-mir-9-2) | 0.000403 | down |
|  | PLA2G2A | hsa-miR-9-5p(hsa-mir-9-1) | 0.000403 | down |
| **MAPK signaling pathway** | FGF7 | hsa-miR-30e-5p(hsa-mir-30e) | 0.012066 | up |
|  | FGF7 | hsa-miR-30c-5p(hsa-mir-30c-2) | 0.026564 | up |
|  | FGF7 | hsa-miR-30c-5p(hsa-mir-30c-1) | 0.025462 | up |
|  | NGF | hsa-let-7e-5p(hsa-let-7e) | 4.10E-08 | up |
| **MAPK signaling pathway** | MAP3K13 | hsa-miR-206(hsa-mir-206) | 0.022004 | down |
| **PI3K-Akt signaling pathway** | LPAR6 | hsa-miR-24-3p(hsa-mir-24-2) | 8.77E-07 | up |
|  | LPAR6 | hsa-miR-24-3p(hsa-mir-24-1) | 9.25E-07 | up |
|  | PDGFC | hsa-let-7d-3p(hsa-let-7d) | 0.020946 | up |
|  | CCND1 | hsa-miR-6087(hsa-mir-6087) | 2.36E-19 | up |
|  | NGF | hsa-let-7e-5p(hsa-let-7e) | 4.10E-08 | up |
|  | PIK3CD | hsa-miR-7704(hsa-mir-7704) | 0.000227 | up |
|  | VTN | hsa-miR-4508(hsa-mir-4508) | 0.001787 | up |
|  | FGF7 | hsa-miR-30e-5p(hsa-mir-30e) | 0.012066 | up |
|  | FGF7 | hsa-miR-30c-5p(hsa-mir-30c-2) | 0.026564 | up |
|  | FGF7 | hsa-miR-30c-5p(hsa-mir-30c-1) | 0.025462 | up |
|  | GNGT2 | hsa-miR-1246(hsa-mir-1246) | 3.44E-06 | up |
| **PI3K-Akt signaling pathway** | PPP2R5C | hsa-miR-221-5p(hsa-mir-221) | 0.007438 | down |
|  | BAD | hsa-miR-1292-5p(hsa-mir-1292) | 0.009353 | down |
| **EGFR-TKI resistance** | PIK3CD | hsa-miR-7704(hsa-mir-7704) | 0.000227 | up |
|  | PDGFC | hsa-let-7d-3p(hsa-let-7d) | 0.020946 | up |
| **EGFR-TKI resistance** | BAD | hsa-miR-1292-5p(hsa-mir-1292) | 0.009353 | down |
| **@@Cell cycle** | CCND1 | hsa-miR-6087(hsa-mir-6087) | 2.36E-19 | up |
